# Classical swine fever virus induces oxidative stress in swine umbilical vein endothelial cells

**DOI:** 10.1186/s12917-014-0279-3

**Published:** 2014-12-02

**Authors:** Lei He, Yanming Zhang, Yanqin Fang, Wulong Liang, Jihui Lin, Min Cheng

**Affiliations:** College of Veterinary Medicine, Northwest A & F University, Yangling, Shaanxi 712100 P.R. China; Animal Disease and Public Security Academician Workstation of Henan province, The Key Lab of Animal Disease and Public security, Henan University of Science and Technology, Luoyang, 471003 P.R. China

**Keywords:** Classical swine fever virus, Oxidative stress, Antioxidant protein, Reactive oxygen species, Inflammatory response

## Abstract

**Background:**

Classical swine fever virus (CSFV) infection causes significant losses of pigs, which is characterized by hemorrhage, disseminated intravascular coagulation and leucopenia. The swine vascular endothelial cell is a primary target cell for CSFV. The aim of this study was to determine the role of CSFV infection in inducing oxidative stress (OS) in vascular endothelial cells.

**Results:**

We demonstrated that CSFV infection induced oxidative stress in swine umbilical vein endothelial cells (SUVECs), characterized by the induction of reactive oxygen species (ROS) production and the elevations of porcine antioxidant proteins thioredoxin (Trx), peroxiredoxin-6 (PRDX-6) and heme oxygenase-1 (HO-1) expression. Furthermore, cyclooxygenase-2 (COX-2), a pro-inflammatory protein related to oxidative stress, was up-regulated while anti-inflammatory protein peroxisome proliferator-activated receptor-γ (PPAR-γ), an important mediator in vascular functional regulation, was down-regulated in the CSFV infected cells. In addition, antioxidants showed significant inhibitory effects on the CSFV replication, indicating a close relationship between CSFV replication and OS induced in the host cells.

**Conclusions:**

Our results indicated that CSFV infection induced oxidative stress in SUVECs. These findings provide novel information on the mechanism by which CSFV can alter intracellular events associated with the viral infection.

## Background

Classical swine fever virus (CSFV), the etiological agent of Classical swine fever (CSF) occurring worldwide, belongs to *Pestivirus* genus of the *Flaviviridae* family [1]. The frequent appearance of CSFV infections across national borders and the considerable socio-economic impact on the pig industry make CSF a notifiable (previously list A) disease by the World Organization for Animal Health (OIE) [2]. The infection of pigs with CSFV strains of high virulence such as shimen strain causes a severe acute disease with high mortality rate, characterized by haemorrhagic diathesis, leucopenia, thrombocytopenia and disseminated intravascular coagulation [3,4]. Since 1990, CSF has been eradicated in many areas of the world through a ‘stamping-out’ slaughter policy. However, the epizootic outbreaks caused considerable financial and sociological impact, and increasing public opposition against stamping-out policies has now led to an increased attention for new vaccines and therapies [5,6]. By now, the pathogenesis of CSFV is still not fully understood and no cure for CSF is presently available apart from symptomatic treatment. Elucidating the mechanism of CSFV pathogenesis will provide new therapeutic targets to treat CSFV infection and its complications.

There is increasing evidence supporting the view that oxidative stress, a deleterious action represented by the elevation of ROS, might play a critical role in the pathophysiology of virus infection [7,8]. Oxidative stress has been implicated in the pathogenesis of various seemingly unrelated viruses, e.g., human immunodeficiency virus [9], human rhinovirus [10], bovine diarrhea virus [11], and hepatitis C virus [12,13]. However, whether oxidative stress occurs on CSFV infected endothelial cells is still unknown, and little information is available on its role in CSFV multiplication. Mounting evidence has emphasized that vascular oxidative stress and increased production of ROS contribute to the mechanism of vascular dysfunction, while it was reported that blood vessel dysfunction plays an important role in the pathogenesis of CSFV [14]. One intriguing possibility is that CSFV through its effect on oxidative stress plays a pathophysiological role in vascular dysfunction. Interestingly, the bioavailability of nitric oxide (NO), a dominant factor responsible for regulating vascular function, was found to be reduced in CSFV infected vascular endothelial cells [15]. And it was also reported to be decreased when oxidative stress and the aberrant expression of ROS occurred [16].

Here, we demonstrated that CSFV infection induced oxidative stress in SUVECs, characterized by the induction of ROS production and associated with the elevations of antioxidant proteins Trx, PRDX-6 and HO-1 expression. Further studies showed that inflammatory related proteins COX-2 and PPAR-γ were also altered in the CSFV infected cells. It was worth noting that antioxidants showed significant inhibitory effects on the CSFV replication, indicating a close relationship between CSFV replication and OS induced in the host cells. The results of these findings have potentially important implications for the mechanism by which CSFV can alter intracellular events associated with the viral infection and the controlling this economically important animal disease.

## Results

### CSFV induces ROS production in SUVECs

**Figure 1:**
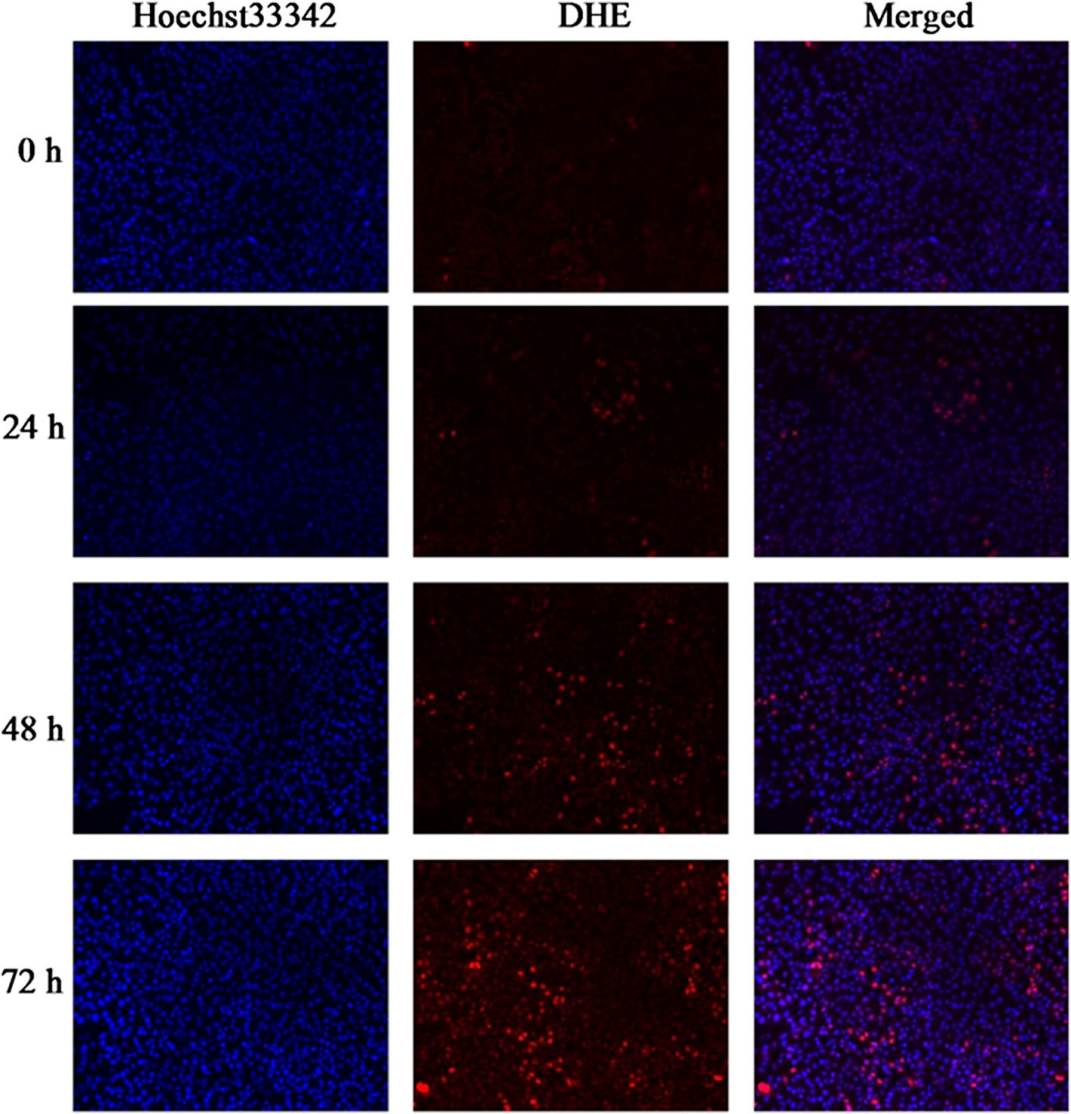
**Up-regulation of intracellular ROS in CSFV infected SUVECs detected by fluorescence microscopy.** The amount of intracellular ROS is increased compared to the control cell in a time depend manner. All SUVEC cells were treated with 1 μM of DHE for 20 min, and then stained by Hoechst33342. Cells dyed red are ROS positive cells, while all the cells nuclei were dyed blue.

**Figure 2:**
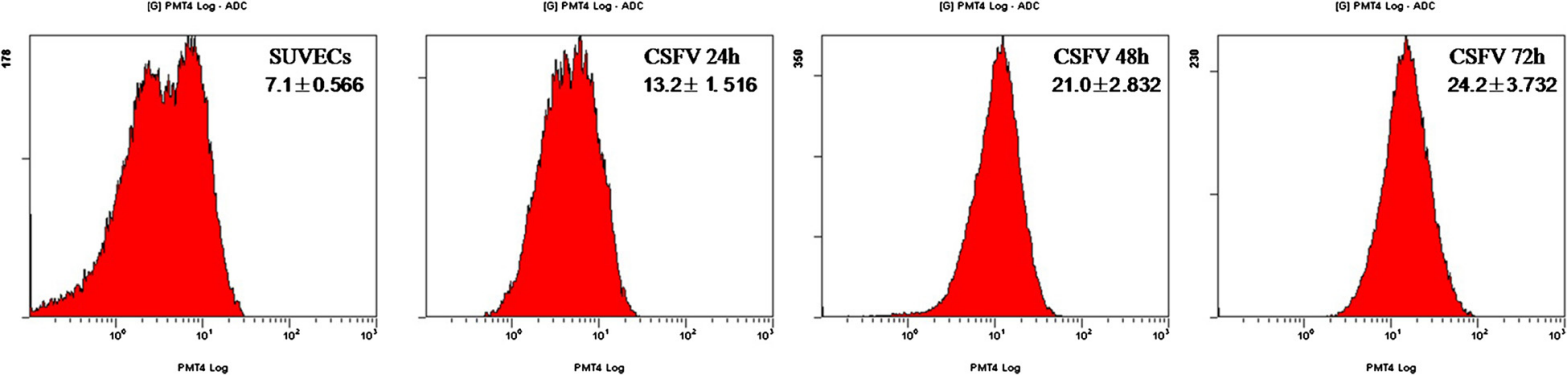
**CSFV infection induces the production of intracellular ROS detected by using flow cytometer.** All SUVEC cells were treated with 1 μM of DHE for 20 min as above. Cells were harvested, and ROS levels were measured with excitation emission at 605 nm. The ratios of mean fluorescent intensity of all the cells are indicated at the right upper corner.

**Figure 3:**
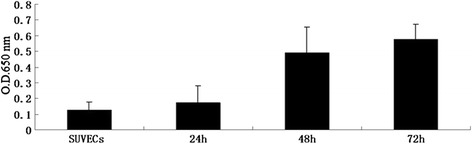
**CSFV infection induces the production of intracellular ROS detected by using superoxide anion in-situ assay.** All the cells were dealt following the instructions to evaluate the ROS level of each sample. OD650 values represent the mean ± SD of three separate experiments performed three times.

The production of ROS, a representative of oxidative stress, was investigated after the infection of CSFV in the SUVECs at 24, 48 and 72 h respectively. Dihydroethidium (DHE) can be oxidized to ethidium by ROS. Ethidium is known to be free to intercalate with DNA in the nucleus, where it emits fluorescence at 605 nm. So in this study, the level of ROS was measured in two ways: fluorescence microscopy and flow cytometry by incubating with DHE. As shown in Figure 1, more cells were dyed red in the CSFV infected cells, indicating that ROS levels were higher compared to the control SUVECs. The level of ROS was increased with prolong cultured time as the level of ROS in SUVECs infected with CSFV at 72 h was significantly higher than 48 h and 24 h. To confirm this observation, the flow cytometry was carried out and results described in Figure 2 clearly show the proportions of cells stained with DHE for CSFV infected cells at 24, 48, and 72 h were 13.2 ± 1.516, 21.0 ± 2.832, and 24.2 ± 3.732 respectively, while for the control cells was 7.1 ± 0.566. It indicated that the production of ROS was increased in CSFV infected cells by a greater extent as compared to the control SUVECs. Furthermore, the ROS levels were also measured by the cell superoxide anion in situ assay. The average values of optical density (OD) at a wavelength of 650 nm per sample were used for statistical analyses. As shown in Figure 3, the CSFV infected cells produced much more ROS leading to a significantly higher in OD values (Figure 3). Taken together, these data suggested that the infection of CSFV is associated with a markedly enhanced expression level of ROS.

### CSFV infection induces elevations of porcine antioxidant proteins expression

**Figure 4:**
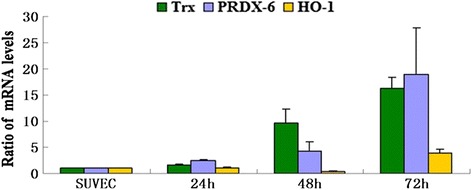
**Effects of CSFV infection on porcine Trx, PRDX-6, and HO-1 expressions in cultured SUVEC cells.** Quantitative real-time RT-PCR analysis of these antioxidant proteins’ mRNA levels was normalized to the corresponding CT value for porcine β-actin mRNA. The basal expression level in SUVEC cells was assigned a value of 1 for each experiment. The data set represents the mean ± SD of experiments repeated three times.

Antioxidant genes’ expression are regulated by the delicate balance between the levels of oxidizing and reducing equivalents of the redox state [17], and the activation of antioxidant proteins is associated with oxidative stress during the viral infection [13,18]. In this assay, the antioxidant proteins thioredoxin (Trx), peroxiredoxin-6 (PRDX-6) and heme oxygenase-1 (HO-1) were examined at 24, 48 and 72 h post-infection. As shown in Figure 4, we found that the mRNA levels of Trx, PRDX-6 and HO-1 were elevated in CSFV infected cells compared to the control cells. Especially, the levels of Trx and PRDX-6 were significantly higher at 72 h post infection, approximately 15-fold higher than in the control SUVECs, and the elevations showed a time-dependent manner after the CSFV infection. The mRNA levels of HO-1 were slightly elevated compared with the controls. These results clearly suggested that CSFV alters the mRNA levels of antioxidants in the SUVEC cells in different levels.

### CSFV infection induces a pro-inflammatory effect on SUVEC

**Figure 5:**
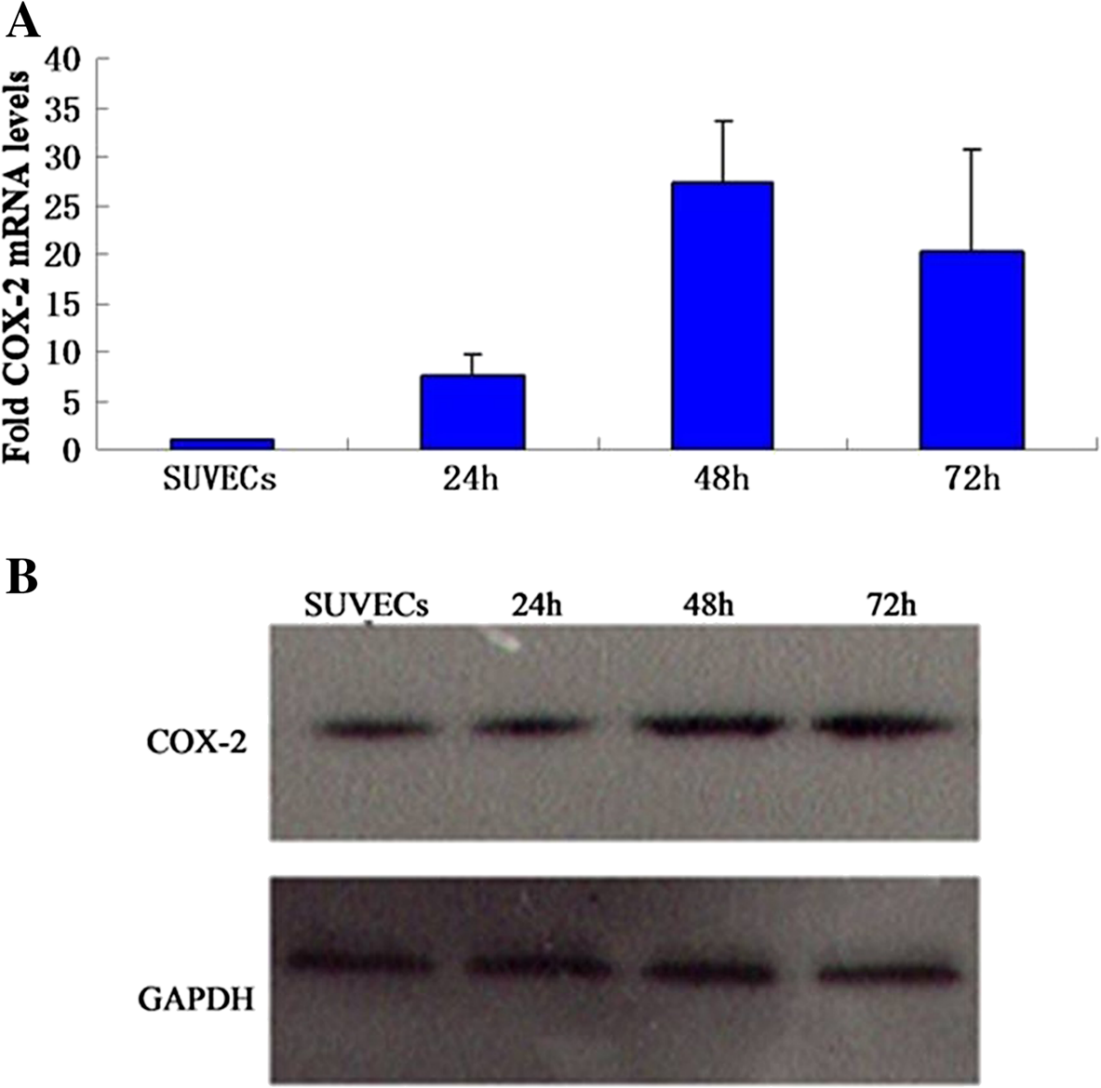
**The effect of CSFV infection on porcine COX-2 expression in cultured SUVEC cells. (A)** Quantitative real-time RT-PCR analysis of COX-2 levels was normalized to the corresponding CT value for porcine β-actin mRNA. **(B)** The level of COX-2 expression was determined by western blot in CSFV infected cells at different points of time.

**Figure 6:**
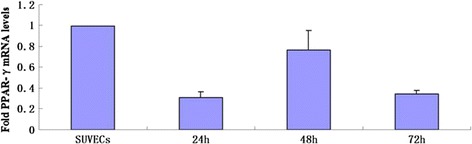
**The effect of CSFV infection on porcine PPAR-γ expression in cultured SUVEC cells.** Quantitative real-time RT-PCR analysis of PPAR-γ levels was normalized to the corresponding CT value for porcine β-actin mRNA. The basal expression level in SUVEC cells was assigned a value of 1 for each experiment. The data set represents the mean ± SD of experiments repeated three times.

Studies have shown that oxidative stress and the inflammation have major roles in vascular diseases. We further examined the effect of CSFV infection on peroxisome proliferator-activated receptor-γ (PPAR-γ) and Cyclooxygenase-2 (COX-2) which are highly expressed in vascular endothelial cells and correlated with both the increased reactive oxygen species (ROS) and the inflammatory response [8]. COX-2 induction in CSFV infected cell was evaluated by quantitative real-time RT-PCR and western blot. CSFV infection could induce an increase on COX-2 mRNA and protein expression as shown in Figure 5A, B, respectively, indicating a role for CSFV in COX-2 induction. To further demonstrate the relationship between CSFV infection and the inflammatory effect, we also examined the alternation of PPAR-γ, which is an anti-inflammatory protein highly expressed in vascular endothelial cells. Quantitative real-time RT-PCR showed that the expression of PPAR-γ in cells infected with CSFV was 5- to 6- fold lower compared with control cells (Figure 6). These observations suggest that the CSFV induces a significant pro-inflammatory effect on SUVECs.

### Inhibitory effects of antioxidants on CSFV viral production

**Figure 7:**
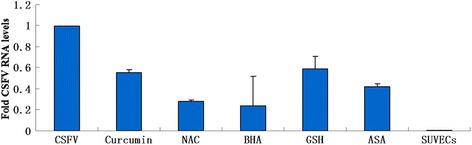
**Inhibition of CSFV RNA replication by antioxidants in cultured SUVEC cells.** The amount of viral genome was measured by real-time RT-PCR, which was normalized to the corresponding β-actin in the same sample. The mean of three repeat experiments performed in triplicate is shown, and error bars represent the SD.

To determine whether the oxidative stress induced by CSFV infection is involved in the viral replication or not, oxidative stress inhibitors glutathione (GSH), N-acetyl-L-cysteine (NAC), butylated hydroxyanisole (BHA) and curcumin, which were reported to counteract the oxygen free radical, were added to the CSFV infected cells. Quantitative real-time RT-PCR was carried out by using the lysates prepared from SUVECs, SUVECs infected with CSFV and CSFV infected cells incubated with GSH, NAC, BHA and curcumin and the COX-2 inhibitor aspirin (ASA) for 72 h, respectively. Results showed that the amount of viral RNA was significantly reduced in the presence of antioxidants in varying degrees (Figure 7), indicating a close association between oxidative stress induced by CSFV and viral replication.

## Discussion

The results from this present study provide evidence that CSFV infection induces oxidative stress and pro-inflammatory response in SUVEC cells and that the inhibition of oxidative stress may significantly decrease viral replication, indicating that oxidative stress is an important factor in CSFV growth in SUVEC cells.

CSFV is found to be highly susceptible to infect vascular endothelial cells [14]. The CSFV replication did not cause direct damage to the vascular endothelial cells; the cell morphology was normal and no CPE or plaque formation was induced in the infected cells [19]. However, the physiological function of vascular endothelial cells (VECs) was remarkably changed post infection [14,15,20]. It was found that vascular endothelial cells infected with CSFV induce tissue factor expression and inhibit apoptosis and interferon synthesis, which may lead to the establishment of long-term infection [14]. In this study, we showed for the first time that the CSFV infection could change the ROS level and the cellular redox state condition in CSFV infected cells, which may be related to the hemorrhaging found in the CSFV infected pigs as it has been suggested that the secretion of ROS from endothelial cells disrupts the haemostatic balance, leading to throm bocytopenia and platelet aggregation followed by haemorrhagic fever [21,22].

Reactive oxygen species (ROS) are produced as a consequence of normal metabolism, which is a double sword depending on their concentration [23,24]. Excess ROS induce oxidative modification of cellular molecules, activate or inhibit protein function, and promote pro-inflammatory factors’ expression [25,26]. Our current observation shows that CSFV infection induces ROS in a time dependent manner. In lines with our finding, the down-regulation of NO production and attenuation of the expression of NO synthase in the CSFV infected porcine macrophages and aortic endothelial cells have been reported [15]. The concentrations of ROS reduce bioactive NO through chemical inactivation, forming toxic peroxynitrite, which in turn can uncouple endothelial NO synthase to form a dysfunctional superoxide-generating enzyme that contributes further to oxidative stress [27,28]. NO is reported to play a key role in vascular responses, such as the capability of leukocytes to adhere to the endothelium, the aggregation of platelets, angiogenesis and the relaxation of vascular smooth muscle cells [29,30]. Therefore, it is tempting to conclude that CSFV induces ROS production, which then inactivates the NO synthase and NO bioavailability results in blood vessel endothelial cells dysfunction.

Modulating superoxide metabolism is one form of defense against excessive ROS production. The antioxidant enzyme Trx PRDX-6 and HO-1 were reported to play critical roles in the clearance of ROS [31-33]. Our results have shown that Trx PRDX-6 and HO-1, which catalyses ROS into water and oxygen, were increased in the CSFV infected cells, indicating that the up-regulation of anti-oxidative stress proteins in host cells as a mechanism to relieve cellular oxidative stress induced by CSFV infection. It is notably to see that Trx is also a known anti-apoptotic factor in endothelial cells [34], and CSFV was reported to protect aortic endothelial cells from pIpC-mediated apoptosis [20]. However, whether the elevation of Trx contributes to the anti-apoptosis effect induced by CSFV infection needs more exploration.

Oxidative stress has been shown to participate in the modulation of inflammatory responses of the many pathological states [35,36]. Cyclooxygenase −2 (COX-2), a major pro-inflammatory mediator, and PPAR-γ, an anti-inflammatory agent, have been evaluated after the infection of CSFV. The up-regulation of COX-2 and down-regulating of PPAR-γ have been observed. These results indicated that CSFV infection play a significant role in inducing inflammation. This observation is consistent with a previous finding that CSFV could stimulate the expression of a number of pro-inflammatory cytokines in endothelial cells [37]. Importantly, the viral RNA replication was reduced by adding COX-2 inhibitor ASA, indicating the elevated level of this protein apparently facilitates the production of virus replication. Other researcher has found that the viral proteins, like NS3, core and NS5A proteins of HCV could activate the production of COX-2. And it has been shown that inactivation of COX-2 was associated with the inhibition of HCV RNA replication [38]. To investigate whether oxidative stress was required by CSFV during replication, Antioxidants PDTC, GSH and NAC were added to the CSFV-infected cells. The RNA level of CSFV infected cells were measured by Real time RT-PCR. The RNA replications of CSFV were reduced in varying degrees, indicating that oxidative stress may be important factor for the virus replication cycle. Consistently, oxidative stress and the inhibitory effect of antioxidants were also observed during different types of viral infections [9,10,39]. However, the present study only investigated the relationship between the viral replication and oxidative stress preliminarily, more studies about antioxidants-mediated inhibition against CSFV replication will be performed in near future.

## Conclusions

In summary, the present results showed that CSFV infection induced oxidative stress by altering host intracellular ROS level and antioxidant status, accompanied with an increase in COX-2 and a reduction of PPAR-γ, contributing to the CSFV induced pro-inflammatory response and vascular dysfunction. The supplemental antioxidants significantly inhibited the viral replication. This study provided novel findings for understanding the molecular mechanisms of CSFV pathogenesis.

## Methods

### Virus and cell culture

Virulent CSFV (shimen strain) was provided by the Control Institute of Veterinary Bio-products and Pharmaceuticals (China) and propagated in the pig kidney cell line PK-15 cells. PK-15 cells were maintained in Dulbecco’s modified Eagle’s medium (DMEM; GIBCO, UK) supplemented with 10% heat-inactivated fetal calf serum (FCS) (Hyclone, China). The established swine umbilical vein endothelial cell line (SUVEC) was cultured as previously described [40]. Briefly, SUVEC cells were cultured at 37°C and 5% CO_2_ in DMEM containing 10% FCS, 50 μg/mL heparin (Sigma-Aldrich, USA), and antibiotics (100 μg/mL streptomycin and 100 U/mL penicillin). The culture medium was replaced every 3 days. All the experiments were approved in accordance with the guidelines of the committee for the Ethics on Animal Care and Experiments at Northwest Agriculture & Forestry University.

### Viral infection

One day before infection, SUVECs were seeded onto 6- well culture plates at a concentration of 2 × 10^5^ cells per well. To infect cells with CSFV, SUVECs (30-50% confluency) were washed twice with PBS and adsorbed with CSFV at a multiplicity of infection (MOI) of 5 for 90 min at 37°C. For control mock-infected cells, an equal amount of uninfected PK-15 cell lysate or PBS was added. The viral inoculum was then removed and the cell monolayers were washed three times with serum-free medium and replaced with DMEM containing 3% FBS. The culture plates were incubated at 37°C with 5% CO_2_ in the humidified incubator.

The effect of GSH, NAC, BHA and curcumin on the amount of virus generated from infected SUVEC cells was examined by applying antioxidants in non-cytotoxic concentrations (50 μM GSH, 30 μM NAC, 300 μM BHA, and 60 μM curcumin, respectively) and the COX-2 inhibitor aspirin (10 μM ASA) after CSFV infection. The effect of these inhibitors on CSFV viral production was observed by Quantitative real-time RT-PCR.

### Fluorescence microscopy

As cellular superoxide especially ROS production could be assessed by oxidation of dihyroethidium (DHE) to ethidium. And the conversion of DHE to ethidium results in red nuclear fluorescence. Alteration of ROS in CSFV infected SUVECs at 24, 48 and 72 h post-infection were detected by using fluorescence microscopy, SUVECs were washed with Hank’s balanced salt solution (HBSS) and incubated with Hoechst 33342 (10 ng/mL) at 37°C for 15 min, and then washed three time with HBSS. SUVECs were incubated with DHE (2 μM; Beyotime, China) at 37°C for 20 min and washed with DMEM without serum. Then the cell images were viewed by fluorescence microscopy (Nikon, Japan).

### Flow cytometry

Alteration of ROS in CSFV infected SUVECs at 0, 24, 48 and 72 h post-infection were also measured by using flow cytometry, SUVECs were incubated with DHE (2 μM; Beyotime, China) for 20 min, and harvested in DMEM of 5% FBS. After being washed two times with PBS, cells finally were suspended in 1 mL of PBS. Superoxide levels were determined by using a Coulter Epics XL flow cytometer with an excitation emission at 605 nm.

### Quantitative real-time RT-PCR

Total RNA was isolated from SUVECs infected with CSFV at 0, 24, 48 and 72 h post-infection by using Trizol reagent (Invitrogen, USA) according to the manufacturer’s protocol and was removed contaminating genomic DNA by being subjected to DNase treatment with RNase-free DNase (Takara Bio, Dalian, China). The cDNA was reverse transcribed from 1 μg of total RNA by using RT primer mix (Takara Bio, Dalian, China). Primers for genes of interest and the housekeeping gene β-actin, whose sequences are shown in Table 1, were designed by using Beacon Designer software program. Quantitative PCR was carried out by using a SYBR ExScript™ RT-PCR Kit (Takara Bio, Dalian, China), according to manufacturer’s instructions. Reactions were performed in an iQ5 Real-Time PCR Detection System (Bio-Rad, USA) under the following conditions: 10 min at 95°C (heat inactivation of reverse transcriptase and activation of taq polymerase), and 42 cycles of 5 s at 95°C, 10 s at 58°C, and 15 s at 72°C (PCR amplification). Data were analyzed according to the Comparative threshold (Ct) method, where the amount of RNA in samples normalized to β-actin and the tests was determined in triplicate.

**Table 1:** **Host gene and CSFV RNA analyzed and primer sets for real-time RT-PCR**

**Genes**	**Forward primer**	**Reverse primer**	**Product length (bp)**
Trx	5′- CAAAGTATTCCAATGTCGTGTTCC-3′	5′ - AGTTCACCCACCTTCTGTCCC -3′	127
PRDX-6	5′ -GTGAGGAGCCCAAGGAAACG-3′	5′ - CCCAACTGGATGGCAAGGTC-3′	73
HO-1	5′-GTCCTCAAGAAGATTGCTCAGAAG-3′	5′-GTCATCTCCAGAGTGTTCATTCG-3′	140
COX-2	5′ -CTTCCTCCTGTGCCTGATGACTG-3′	5′-GGTCCTCGCTTCTGATCTGTCTTG-3′	200
PPAR-γ	5′-TGACAGGAAAGACCACAGACAAAT-3′	5′-GGGTGATGTGTTTGAACTTGATT-3′	96
CSFV	5′- TCAACCGAAGAAATGGGAGATG-3′	5′ - TCCACCCTATTGGGCAGACA-3′	133
β-actin	5′ -CGTCCACCGCAAATGCTTC-3′	5′ -AACCGACTGCTGTCACCTTCAC-3′	217

### Western blot

The SUVECs extracts were prepared by washing cells with PBS, harvested by scraping and then suspended in 1 mL PBS. After clarification by centrifugation at 3 000 g for 5 min at 4°C, the cells were resuspended in cell lysis buffer (50 mM Tris–HCl, 5 mM EDTA, 150 mM NaCl, 0.1% NP-40, 0.5% deoxycholic acid, 1 mM sodium orthovanadate, 100 μg/mL PMSF and protease inhibitors) and centrifuged at 15 000 g for 30 min at 4°C. Equal amounts of total protein (15 μg/lane) were separated by 12% sodium dodecyl sulphate polyacrylamide gel electrophoresis (SDS-PAGE) and transferred onto a 0.2-mm PVDF membrane (Millipore, USA) using a Trans-Blot SemiDry transfer device (BioRad). Membranes were blocked for 1 h at room temperature with 5% skim milk in TBST buffer [20 mM Tris–HCl (pH 7.5), 50 mM NaCl, and 0.05% Tween 20]. For immunodetection, the membranes were incubated with mouse anti-COX-2 (Abcam, UK) or mouse anti-porcine GAPDH (Genscript, USA) antibody overnight at 4°C with 5% skim milk in TBST buffer and incubated at 37°C with horseradish peroxidase-conjugated goat anti-rabbit antibody for 1 h at 37°C. Between each incubation step, membranes were washed with TBST buffer. The protein bands were visualized by enhanced chemiluminescence methods as per the manufacturer’s instructions (Millipore, USA).

## Abbreviations

CSFV: Classical swine fever virus
OS: Oxidative stress
SUVECs: Swine umbilical vein endothelial cells
VECs: Vascular endothelial cells
ROS: Reactive oxygen species
Trx: Thioredoxin
PRDX-6: Peroxiredoxin-6
HO-1: Heme oxygenase-1
COX-2: Cyclooxygenase-2
PPAR-γ: Peroxisome proliferator-activated receptor-γ
NO: Nitric oxide
GSH: Glutathione
NAC: N-acetyl-L-cysteine
BHA: Butylated hydroxyanisole
ASA: Aspirin
FCS: Fetal calf serum
HBSS: Hank’s balanced salt solution
